# Heterologous oligonucleotide microarrays for transcriptomics in a non-model species; a proof-of-concept study of drought stress in *Musa*

**DOI:** 10.1186/1471-2164-10-436

**Published:** 2009-09-16

**Authors:** Mark W Davey, Neil S Graham, Bartel Vanholme, Rony Swennen, Sean T May, Johan Keulemans

**Affiliations:** 1 Laboratory for Fruit Breeding and Biotechnology, Department of Biosystems, Katholieke Universiteit Leuven, Box 2747, Willem De Croylaan 42, B-3001, Heverlee, Leuven, Belgium; 2 Nottingham *Arabidopsis *Stock Centre, School of Biosciences, University of Nottingham, Sutton Bonington Campus, Loughborough, LE12 5RD, UK; 3 Department of Plant Systems Biology, VIB, and Department of Molecular Genetics, Universiteit Gent, Technologiepark 927, B-9052 Gent, Belgium; 4 Department of Biosystems, Katholieke Universiteit Leuven, Kasteelpark Arenberg 13 Box 2455, B - 3001 Leuven, Belgium

## Abstract

**Background:**

'Systems-wide' approaches such as microarray RNA-profiling are ideally suited to the study of the complex overlapping responses of plants to biotic and abiotic stresses. However, commercial microarrays are only available for a limited number of plant species and development costs are so substantial as to be prohibitive for most research groups. Here we evaluate the use of cross-hybridisation to Affymetrix oligonucleotide GeneChip^® ^microarrays to profile the response of the banana (*Musa *spp.) leaf transcriptome to drought stress using a genomic DNA (gDNA)-based probe-selection strategy to improve the efficiency of detection of differentially expressed *Musa *transcripts.

**Results:**

Following cross-hybridisation of *Musa *gDNA to the Rice GeneChip^® ^Genome Array, ~33,700 gene-specific probe-sets had a sufficiently high degree of homology to be retained for transcriptomic analyses. In a proof-of-concept approach, pooled RNA representing a single biological replicate of control and drought stressed leaves of the *Musa *cultivar 'Cachaco' were hybridised to the Affymetrix Rice Genome Array. A total of 2,910 *Musa *gene homologues with a >2-fold difference in expression levels were subsequently identified. These drought-responsive transcripts included many functional classes associated with plant biotic and abiotic stress responses, as well as a range of regulatory genes known to be involved in coordinating abiotic stress responses. This latter group included members of the ERF, DREB, MYB, bZIP and bHLH transcription factor families. Fifty-two of these drought-sensitive *Musa *transcripts were homologous to genes underlying QTLs for drought and cold tolerance in rice, including in 2 instances QTLs associated with a single underlying gene. The list of drought-responsive transcripts also included genes identified in publicly-available comparative transcriptomics experiments.

**Conclusion:**

Our results demonstrate that despite the general paucity of nucleotide sequence data in *Musa *and only distant phylogenetic relations to rice, gDNA probe-based cross-hybridisation to the Rice GeneChip^® ^is a highly promising strategy to study complex biological responses and illustrates the potential of such strategies for gene discovery in non-model species.

## Background

Bananas and plantains are large herbaceous monocots from the genus *Musa *of the family *Musaceae*. The vast majority of cultivated bananas are hybrids derived from natural inter- and intraspecific crosses between two diploid wild species, *Musa acuminata *(designated by genome A) and *M. balbisiana *(designated by genome B) [1]. These diploid, triploid or tetraploid hybrids are of great economic importance in sub-Saharan Africa, South and Central America and Asia, where they are a staple food for an estimated 400 million people. Although *Musa *spp. are mainly cultivated in tropical and subtropical regions, rain water supply is often far from uniform so that more or less-pronounced dry seasons exist which have an impact on fruit quality and yields. To date there have been few comparative studies on *Musa *drought stress tolerance, but field observations suggest that B-genome confers greater tolerance to drought than the A-genome [2,3].

Plants display a wide range of overlapping responses to biotic and abiotic stresses, and the diversity of physiological, biochemical and molecular strategies adopted during adaptation to unfavourable environmental conditions (including drought), creates particular problems for the scientist wishing to study and understand them [4-6]. Non-biased, 'systems-wide' approaches such as transcriptomics and microarray RNA-profiling are well-suited to the analysis of this type of problem and have provided many insights into the pathways of (a)biotic stress response and adaptation in a variety of model plant systems [7-11]. However, while cDNA and oligonucleotide microarrays are now routinely used for transcriptomic analyses in plants, the number of species for which commercial microarrays are available is very limited. For non-model plant species such as *Musa *where little sequence information is available, microarray development costs are so substantial as to be prohibitive for most groups working in the area. However it has been recently demonstrated that commercially available high-density oligonucleotide microarrays from closely related, heterologous species can be used to probe the transcriptomes of non-model plants. For example the *Arabidopsis *Affymetrix ATH-1 Genome Array has been used to study the transcriptome of *Brassicacea *species such as *Arabidopsis halleri *[12,13], *Thlaspi caerulescens *[14,15], *Thelungiella halophila *[16,17], *Brassica oleracea *[18] and *Brassica napus *[19]. In addition a tomato array has also been used to study fruit ripening and development in tomato, aubergine and pepper [20], as well as in potato [21].

In spite of the economic and social importance of banana and plantains and its relatively small haploid genome size (amongst the monocots) of 560 - 600 Mbp [22,23], little sequence information is publicly accessible. Available published data includes the complete sequencing of two BAC clones from a wild diploid banana cultivar [24], the analysis 6,252 BAC end-sequences [25] and recently the sequencing of 13 BAC clones and the analysis of 17 BAC clones collectively containing a total of 443 predicted genes [26]. In addition, 5,292 unique foliar 24 bp transcript sequences were sequenced and identified in the cultivar *Musa acuminata *following SuperSAGE [27]. The only transcriptomics study published to date however, describes the creation of cDNA libraries from *Musa *plants subjected to temperature stress. This lead to the identification of 2,286 high-quality sequences, of which 715 where considered to be full-length cDNA clones representing a set of 149 unique genes. [28]. The aim of the work presented here therefore was to determine the feasibility of using commercial heterologous oligonucleotide microarrays to probe the *Musa *transcriptome in response to drought stress in the relatively drought-tolerant *Musa *triploid cultivar 'Cachaco' (ABB genome composition). The use of highly standardized, commercial Affymetrix GeneChips^® ^arrays allows comparisons to be made with probe-set data sets from other plant species and helps to reduce cross-laboratory errors. Because of the phylogenetic relationship between target and probe, this work with *Musa *necessarily involves cross-hybridising to species that are evolutionarily much more distant than has previously been attempted.

Clearly the success in identifying differentially-regulated transcripts via cross-hybridisation depends on the degree of similarity between the target and probe sequences. With the Affymetrix GeneChip^® ^Genome Array, the expression level of any one particular gene transcript is generally calculated as the mean of the expression levels of the 11 - 16 perfect match (PM) 25 bp probe-pairs that make up the 'probe-set' for each individual transcript. Therefore inefficient hybridisation of transcripts from the test species of interest to the GeneChip^® ^target probes, due for example to the presence of sequence polymorphisms, will affect the overall signal calculated across a probe-set, reducing the number of 'present' calls, and as a consequence the mean strength of the signal for that transcript's probe-set [14,29]. To eliminate non-hybridising probes, Hammond *et al*, first carried out a cross-hybridisation with gDNA from the test organism. The results allow a 'probe mask' to be created by which weak- or non-hybridising probes can be discarded without discarding the signal for the entire probe-set for a particular transcript in subsequent analyses [18]. The disadvantage of this method is that it can realistically only be used on microarray platforms that contain multiple probes per gene. Applying it to arrays with only one probe per gene (cDNA microarrays) will result in a much lower number of genes for which expression levels can be determined. An alternative approach is to create a 'Global Match File' or a list of 'highly reliable' GeneChip^® ^probe-sets for analysis based on alignment to EST-derived clusters/singletons. This latter heterologous hybridisation approach has been used to study cold-induced sweetening of potato tubers using the Affymetrix tomato microarray [21], but its success depends on the availability of extensive EST sequences to create the Global Match File, and these are not yet available for *Musa*. Interestingly, gDNA-based probe selection has recently been shown to also improve the analysis of differentially expressed genes in homologous species, again by accounting for the impact of differences in the physical hybridisation characteristics of individual probes on probe-set signal intensities [18,30].

Here we report on work to evaluate the use of commercial, high-density Affymetrix Rice and *Arabidopsis *ATH-1 GeneChip^® ^microarrays to analyse complex plant responses in a distantly-related, non-model plant species. Using a gDNA probe-based cross-hybridisation approach we were able to rapidly profile the response of *Musa *transcriptome to chronic drought stress, and to identify a range of structural and regulatory *Musa *gene-homologues previously found to be associated with the water-deficit response in other (model) plants. Further, despite the large phylogenetic difference between *Musa *target and the Rice or *Arabidopsis *probes, comparisons to publically available transcriptome-profiling experiments identified a range of common drought-responsive genes, supporting the assigned *Musa *gene identities and descriptions. This approach outlines the potential of this strategy for the characterization of stress-resistance in banana and plantain varieties for which relatively little sequence information is currently available.

## Results and Discussion

Microarrays for a number of plant species are now commercially available. Of these, the *Arabidopsis *ATH-1 GeneChip^® ^Genome Array is probably the best-characterised/annotated, containing over 22,500 probe-sets representing ~24,000 genes . Similar high density oligonucleotide Affymetrix microarrays also exist for important monocotolydenous crops, including maize, wheat, barley and sugar cane, but the Affymetrix Rice Genome Array contains many more probe-sets (~57,000), and covers a larger portion of the transcriptome than these other arrays.

### gDNA cross-hybridisations and creation of probe mask (.cdf) files

**Figure 1:**
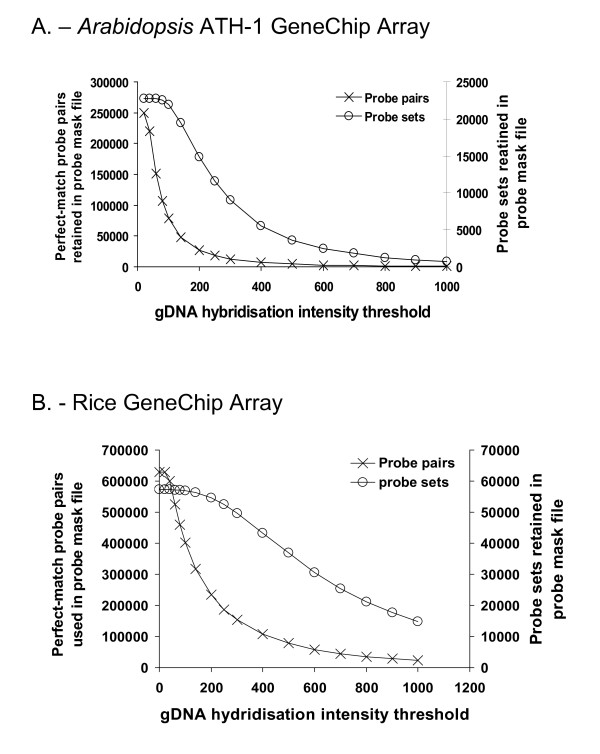
**Impact of hybridisation intensity thresholds on the number of probes and probe-sets retained in the probe mask following hybridisation of Musa gDNA to A**. Affymetrix Arabidopsis ATH-1 GeneChip^® ^and B. Affymetrix Rice GeneChip^®^

Each individual gene transcript on the Affymetrix Rice and *Arabidopsis *ATH-1 GeneChip^® ^microarray, is represented by a set of eleven, 25 bp 'probe-pairs' that make up a 'probe-set', and it is the average hybridisation intensity across this probe-set that is used to calculate expression levels for that gene. To identify and eliminate oligonucleotide GeneChip^® ^probes with low or no-hybridisation to *Musa *transcripts, we used the gDNA probe-masking strategy of Hammond *et al *[14,30]. Here, 'Cachaco' genomic DNA (gDNA) was first hybridised to both the *Arabidopsis *ATH-1 and the Rice GeneChip^® ^Genome Array using standard Affymetrix hybridisation protocols. From the resultant gDNA cell intensity file (.cel) file, perfect match probe-pairs showing a high hybridisation signal to *Musa *gDNA were selected and probe masks (a chip definition file, or .cdf file) then created to exclude non-hybridising probe-pairs within each transcript probe-set. The hybridisation intensity threshold for probe exclusion is set arbitrarily and ranges from 0 (no probe selection) to 1000. The minimum requirement for a probe-set to be called 'present' therefore is hybridisation to at least one probe-pair within that probe-set. [14,18]. The resultant probe mask (.cdf) files are then used to analyze gene expression levels following cross hybridisation of *Musa *RNA samples to the GeneChip^®^. The influence of hybridisation intensity thresholds on probe and probe-set retention for both the ATH-1 and Rice Genome Arrays following cross hybridisation to *Musa *gDNA is shown in Figure 1

As can be seen in Figure 1, the number of probes retained decreases rapidly with increasing hybridisation intensity threshold (CDF) values, while the number of probe-sets (transcripts) retained decreases relatively slowly and only at higher CDF values. As a result the number of probe-sets retained for transcriptome analysis within a probe mask file remains high, even as more probes are excluded. For example after cross hybridisation to the Rice Genome Array, a probe mask created at a CDF value of 550, excludes 87.6% of the total PM probes, even though some 64.4% of the probe-sets are still represented by at least one probe-pair and can be used for subsequent transcriptome profiling. This is similar to the results obtained by Hammond *et al *following cross-hybridisation of *B. Oleracea *and *Thlaspi arvense sp*. gDNA to the ATH-1 Genome Array [14,18]. In our case the average number of probes present per probe-set at an optimum CDF value of 550 was 2.0, with a total of 16,416 probe-sets being represented by a single probe-pair.

Following hybridisation of 'Cachaco' gDNA to the ATH-1 Genome Array, only 1,321 probe-sets were retained at a CDF value of 0 (no filtering). This corresponds to ~5.7% of the total available *Arabidopsis *transcriptome. However at a CDF value of 500, 3,594 or 15.8% of the available *Arabidopsis *transcriptome was retained. Unsurprisingly, hybridisation of 'Cachaco' gDNA to the Rice Genome Array produced far better results, with over 78,000 probe-pairs corresponding to over 36,000 probe-sets (64.4%) being retained at a CDF value of 550. These results therefore broadly confirm the phylogenetic relationships between *Musa *and either *Arabidopsis *or *Oryza *with current estimates indicating that *Musa *and *Oryza *diverged at the level of the order *Zingiberalae *some 65 Mya, while the closest evolutionary link between *Arabidopsis *and *Musa *occurs at the point that the eudicots diverged some 145 - 208 Mya [31]. Interestingly the existence of regions of microsynteny between rice, *Arabidopsis *and *Musa *have recentlybeen demonstrated [26]. For example, even though *Musa *genes generally have a GC structure more closely resembling rice than *Arabidopsis*, out of 443 *Musa *predicted proteins, 268 and 224 had hits with an E-value threshold of 1e^-10 ^to rice and *Arabidopsis *respectively.

On the basis of the gDNA hybridisation results presented here and the closer synteny of *Musa *gene sequences to rice rather than *Arabidopsis *[26,31], transcriptomics experiments were carried out with the Rice GeneChip^® ^array rather than with the Arabidopsis ATH-1 GeneChip Array.

### RNA hybridisations - measuring transcript abundance

**Figure 2:**
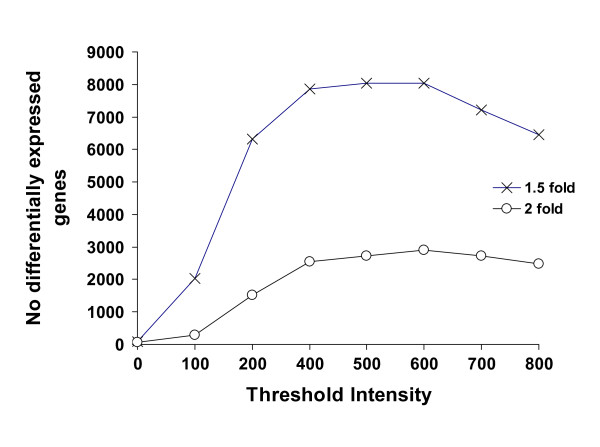
**The influence of the gDNA hybridisation intensity thresholds used to create the gDNA probe mask (.cdf) files, on identification of differentially-regulated genes, following hybridisation of labeled Musa gDNA to the Affymetrix Rice GeneChip^® ^arrays**.

The response of the *Musa *transcriptome to chronic drought stress was examined by challenging Rice Genome Arrays with 'Cachaco' foliar RNA, isolated and pooled from plants subjected to either 3 weeks water-deficit conditions or to control conditions, as described in 'methods'. The ability to detect differentially-expressed transcripts depends upon the hybridisation intensity threshold cut off values (CDF values) used to create the probe mask (.cdf) files as summarised in Figure 2. As can be seen, there is a significant loss in the number of probe-sets retained at higher CDF values, but the number of differentially-regulated genes identified is still greater than when no probe-masking is used [14,18]. Indeed at the optimum CDF value, over 40-fold more >2-fold drought-responsive transcripts are identified than is the case without masking (CDF = 0).

As shown in Figure 2, the maximum number of differentially-expressed transcripts was detected using a CDF value of 550. At this cut off level, a total of 5,292 (RMA normalized expression value >20) of the maximal 33,696 probe-sets hybridised to *Musa *RNA at levels above background suggesting that ~16% of the available *Musa *genome was being expressed at any one time. Based on a single biological replicate, 2,910 transcripts of these transcripts displayed a >2-fold difference in expression levels in response to drought conditions, representing ~8% of the total available transcriptome [see Additional file 1].

This value is similar to values reported in other transcriptomic studies of plant drought stress-responses. For example Talame *et al *[32] using a cDNA microarray found 173 barley genes out of the 1654 genes tested (~10%), to be differentially regulated following 7 days dehydration stress and Seki *et al *[33], found that 277 genes from a cDNA array of 7,000 genes (~4%), were drought-responsive in *Arabidopsis*. Clearly these responses are dependent on the tissue, the severity and length of exposure to stress, as well as the sampling time points [33,34].

Of the 2,910 differentially-regulated putative drought-responsive transcripts detected, 1,671 were up-regulated (57.4%), with the most responsive transcript (Affymetrix probe-set ID Os.53488.1.S1_at; GenBank AK072922, 'expressed protein'), increasing 15.7-fold in relation to the control. The remaining 1,239 (42.6%) transcripts were down-regulated, with the transcript coding for a putative chlorophyll *a *apoprotein (Affymetrix probe-set ID Os.26751.1.S1_s_at; GenBank AK062299), being down-regulated over 100-fold.

#### Gene Ontologies

The rice descriptions and annotations for these 2,910 drought-responsive *Musa *transcripts were obtained from the list of Rice GeneChip^® ^probe-set i.d.s using the HarvEST software available at . From this list, 1,512 genes (54.9%) could also be assigned an *Arabidopsis *annotation using the same software. These *Arabidopsis *annotations were then used for gene ontology (GO) classifications using the 'Gene Ontology' function at the TAIR website . Results are summarised in table 1.

**Table 1:** Functional gene annotations ("Biological Processes") of *Musa *foliar transcripts identified as being >2-fold differentially regulated in response to 3-weeks chronic drought stress.

**Functional Category** **Biological Processes**	**Gene Count**						
	
	**>2-fold** **total**	**%**	**p value**	**>2-fold up**	**%**	**>2-fold down**	**%**
other cellular processes	588	22.3	3.5 × 10^-5^	347	21.9	265	22.6

other metabolic processes	574	21.8	0.0088	335	21.2	265	22.6

unknown biological processes	368	14.0	1.05 × 10^-21^	242	15.3	141	12.1

protein metabolism	226	8.6	0.028	143	9.0	98	8.4

response to stress	132	5.0	0.017	82	5.2	63	5.4

response to abiotic or biotic stimulus	137	5.2	0.032	84	5.3	61	5.2

developmental processes	104	4.0	7.73 × 10^-7^	59	3.7	47	4.0

transport	113	4.3	0.031	68	4.3	48	4.1

other biological processes	102	3.9	0.004	69	4.4	37	3.2

cell organization and biogenesis	91	3.5	0.028	54	3.4	43	3.7

signal transduction	66	2.5	0.051	43	2.7	26	2.2

transcription	72	2.7	0.0003	38	2.4	35	3.0

electron transport or energy pathways	37	1.4	7.38 × 10^-6^	8	0.5	29	2.5

DNA or RNA metabolism	21	0.8	0.078	11	0.7	12	1.0

**SUM**	**2631**	**100**		**1583**	**100**		**100**

As shown in table 1, the distribution of >2-fold differentially-regulated transcripts between GO classes is similar for both up- and down-regulated transcripts, with in both cases the largest proportion (~67%) being represented by 'other biological', 'other metabolic' or 'other cellular' groups. However in agreement with models for plant water-deficit stress responses, there was a general up-regulation of genes involved in 'abiotic stress' responses, 'transport' and 'cell organisation and biogenesis', and a down-regulation of transcripts associated with 'electron transport and energy processes'. Of the functional genes, 137 (5.2%) were assigned to the group 'response to abiotic or biotic stimulus' and 85 (5.0%) to the category 'response to stress'.

Because of their potential importance for crop improvement programs, there is much interest in identifying regulatory genes such as transcription factors (TFs) which are responsible for the coordinate regulation of the large gene sets involved in stress response and adaptations. The group of drought-responsive *Musa *transcripts included 72 genes (2.7%) involved in 'transcription' processes. Within this group there are 8 members (homologues) of the AP2/EREBP TF family, including two DREB family TFs (TAIR: At1g19210, At1g78080). Members of this family have been implicated both in the regulation of both dehydration and cold stress adaptation [35]. Additionally there are homologues of 9 NAC-domain transcription factors (TFs), as well as 4 bZIPs, 8 bHLHs and 7 MYB-domain TFs. Again, members of these families have all been demonstrated to be involved in dehydration and/or temperature-stress-responsive gene expression and to have binding sites in the promoter region of stress-inducible *Arabidopsis *and/or rice genes - for reviews see [5,6,36]. In addition, and in common with results from other groups, we also observed the induction of TFs associated with biotic stress responses including for example several up-regulated members of the WRKY superfamily (TAIR:At5g56270, At4g01720, At4g01250, At5g01900) a family of genes known to be involved in the regulation of plant pathogen responses and senescence - for review see [37]. The differential regulation of transcripts involved in both biotic and abiotic stress-response pathways is a characteristic of plant (drought) stress responses in part due to the role of stress-hormone signalling in coordinating common, overlapping responses and can lead to the phenomenon of 'cross-tolerance' [38,39]. For example the early gene responses to both drought and salt stress are nearly identical, and members of the DREB TF family are involved in the ABA-independent transduction of both drought and cold signals [4-6].

GOs for the list of *Musa *putative drought-responsive genes were also assigned using the Genespring GX 7.3 software (Agilent Technologies, USA), based on Affymetrix NetAffx annotation , the HarvEST rice transcript annotations as well as the HarvEST annotations for the equivalent *Arabidopsis *homologues. From these data, the GO classes that were over-represented in response to drought stress relative to the entire genome were identified. From this list of GO classes, the expression of genes involved in photosynthesis and phenylpropanoid metabolism were amongst the classes most greatly affected by drought conditions.

#### Visualising cellular responses to drought

**Figure 3:**
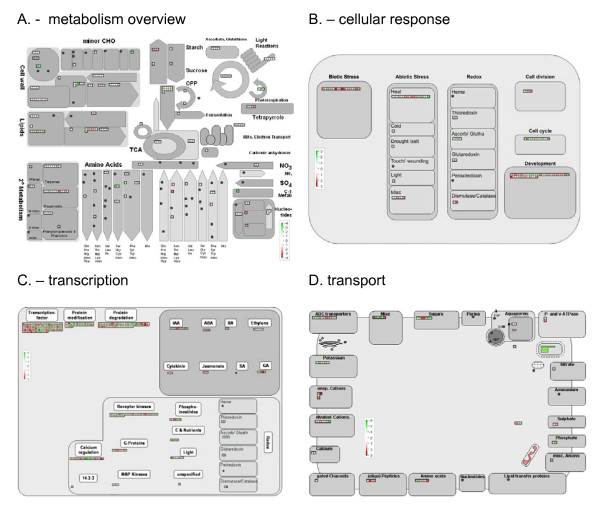
**Overview of the changes in *Musa *foliar transcript levels in response to 3 weeks chronic drought-stress, for genes associated with A) metabolism B) cellular response C) transcription D) transport**. Red and green represent a decrease and an increase of expression respectively, relative to control plants. *Arabidopsis *gene annotations for the list of >2-fold differentially expressed genes obtained from the HarvEST website.

To visualize the effects of drought stress in *Musa *on a cellular level, the Affymetrix *Arabidopsis *codes for the list of >2-fold differentially expressed homologues on the Rice GeneChip were used to run the 'MapMan' software [40]. Although only 1,430 of the 2,910 rice transcripts had an *Arabidopsis *code, the results nonetheless generate an overview of *Musa *cellular responses to drought. As shown in Figure 3, the *Arabidopsis *homologues of the drought-responsive sensitive *Musa *transcripts map to a range of pathways and functions consistent with plant abiotic stress responses [see Additional file 2]. These include 'biotic' (bin = 20.1; p = 0.1908), 'abiotic stress' (bin = 20.2; p = 0.7611), 'hormone response', 'development' (bin = 33; p = 0.5379) and 'cell wall metabolism'(bin = 10.1; p = 0.3212). Although these mappings to the pathways are not statistically significant, probably due to the cross-species nature of the analysis, they do indicate the range of processes that may be involved in the response of *Musa *to drought stress.

Interestingly only one *Arabidopsis *homologue (TAIR: At3g05890) was present in the drought/salt-responsive BIN. Rather, the majority of 'stress-related' transcripts were found in the 'heat', 'biotic stress', and 'development' BINs (Figure 3b). This again reflects the overlapping and redundant nature of plant responses to water-deficit stress. Other functional transcripts of interest were a number of homologues classified in 'redox responses', including for example a cytoplasmic Cu-Zn SOD (TAIR: At1g08830), known to be up-regulated in response to oxidative stress [41], as well as an up-regulated dehydroascorbate reductase transcript (TAIR: At1g75270, DHAR2), which is important for the maintenance of L-ascorbate pools, and which again is central to general stress adaptation responses [42,43]. Interestingly, two transcripts encoding for a putative trehalose-6-phosphate synthase (TPS) were also up-regulated 2.2 and 2.4-fold respectively, and TPS overexpression has been shown to confer drought tolerance in several plant species [44].

#### Rice drought QTLs

To help assess the biological relevance of these results, the list of drought-responsive *Musa *transcripts was compared to the list of genes underlying known QTLs for abiotic stresses in rice, obtained from the Gramene website . Of the 2,910 differentially regulated *Musa *genes, 52 co-localised to a total of 6 QTLs responsible for either drought or cold-response. These results and the functional annotations of all of the identified putative drought-responsive genes present at each QTL are summarised in table 2.

**Table 2:** List of rice homologues of drought responsive *Musa *transcripts that co-localise with known QTLs for 'drought' and 'cold' in rice.

**Rice Abiotic Stress QTLs**	**Rice Homologues of Drought Responsive *Musa *transcripts**		***Arabidopsis *Accession**	**Normalized Expression levels**
			
	**> 2 fold expressed genes**	**Description**		
**QTL-1: cold tolerance** "AQAV003" Chromosome 1 5556378 -7443721 bp Os01g10504 - Os1g13360	Os01g10490*	putatitve protein meiosis 5	AT2G42840.1	**2.56**
	
	Os01g10700	D-mannose binding lectin family protein	AT4G32300.1	**2.04**
	
	Os01g10830	carnitine racemase like protein, putative, expressed	AT4G14430.1	**2.19**
	
	Os01g11010	peptide-N4-asparagine amidase A, putative, expressed	AT3G14920.1	**0.50**
	
	Os01g11020	conserved hypothetical protein	AT2G29210.1	**2.14**
	
	Os01g11120	expressed protein		**2.03**
	
	Os01g11250	potassium channel KAT1, putative, expressed	AT5G46240.1	**0.24**
	
	Os01g11340*	CYP710A1, putative, expressed	AT2G34500.1	**2.53**
	
	Os01g11620*	esterase precursor, putative, expressed	AT5G26780.1	**0.15**
	
	Os01g11720	Transposable element protein, putative, Transposase_24		**0.40**
	
	Os01g12070	endoglucanase 1 precursor, putative, expressed	AT1G64390.1	**0.49**
	
	Os01g12280	protein dimerization, putative, expressed	AT1G79740.1	**2.34**
	
	Os01g12310	histone deacetylase, putative	AT4G38130.1	**2.36**
	
	Os01g12330	expressed protein	AT5G59950.2	**2.42**
	
	Os01g12570	3-methyl-2-oxobutanoate hydroxymethyltransferase, putative, expressed	AT3G61530.1	**0.40**
	
	Os01g12740	cytochrome P450 71A1, putative, expressed	AT3G48290.1	**2.27**
	
	Os01g12810	leaf protein, putative, expressed	AT5G25630.1	**4.02**
	
	Os01g13050	hypothetical protein	AT1G30900.1	**2.56**

**QTL-2: Drought susceptibility** "AQA045/CQ148" Chromosome 4 413619452 - 13619571 bp Os04g23890	Os04g23890	phototropin-1, putative, expressed	AT5G58140.3	**2.23**

**QTL-3: drought tolerance** "AQAN001" Chromosome 5 27321395 - 28590239 bp Os05g47820 - Os05g50000	Os05g47940	transposon protein, putative, unclassified, expressed	AT3G19430.1	**2.15**
	
	Os05g47980	ATP synthase beta chain, mitochondrial precursor, putative, expressed	AT5G08670.1	**2.12**
	
	Os05g48010	anthocyanin regulatory C1 protein, putative, expressed	AT5G57620.1	**0.27**
	
	Os05g48290	T-complex protein 1 subunit beta, putative, expressed	AT5G20890.1	**4.42**
	
	Os05g48320	60S ribosomal protein L37a, putative, expressed	AT3G60245.1	**2.30**
	
	Os05g48600	galactosylgalactosylxylosylprotein 3-beta-glucuronosyltransferase 1 putative, expressed	AT1G27600.1	**0.44**
	
	Os05g48750	3-deoxy-manno-octulosonate cytidylyltransferase, putative, expressed	AT1G53000.1	**2.00**
	
	Os05g48790	expressed protein		**2.28**
	
	Os05g49070	expressed protein	AT1G71180.1	**0.27**
	
	Os05g49160*	negatively light-regulated protein, putative, expressed	AT5G64130.1	**2.82**
	
	Os05g49320	50S ribosomal protein L12-1, chloroplast precursor, putative, expressed	AT3G27830.1	**2.69**
	
	Os05g49510	expressed protein	AT2G40070.1	**2.45**
	
	Os05g49530	F-box domain containing protein	AT1G16610.1	**0.31**
	
	Os05g49610	expressed protein	AT5G62990.1	**0.22**
	
	Os05g49880	malate dehydrogenase, mitochondrial precursor, putative, expressed	AT1G53240.1	**2.03**
	
	Os05g49910	expressed protein	AT2G25605.1	**0.40**

**QTL-4: cold tolerance** AQDU003 Chromosome 8 ]20519931 - 21012373 bp Os08g33190 - Os08g33790	Os08g33390	HECT domain and RCC1-like domain-containing protein 2, putative, expressed	AT5G16040.1	**2.21**

**QTL-5: cold tolerance** "CQP8" Chromosome 11 1491600-1492616 bp Os11g03794	Os11g03794	Aladin, putative, expressed	AT3G56900.1	**3.10**

**QTL-6: drought** **susceptibility** "AQA046/CQA149" Chromosome 12 26017330-27488769 bp Os12g42020-Os12g44490	Os12g42180	50S ribosomal protein L14, putative, expressed	AT5G46160.1	**2.05**
	
	Os12g42250	expressed protein	AT1G68360.1	**0.43**
	
	Os12g42380*	expressed protein	AT1G31810.1	**0.38**
	
	Os12g42510	expressed protein	AT1G31810.1	**0.38**
	
	Os12g42560	hypothetical protein		**0.46**
	
	Os12g42570	expressed protein	AT3G11760.1	**0.22**
	
	Os12g42884	5-methyltetrahydropteroyltriglutamate-homocysteine methyltransferase, putative, expressed	AT5G17920.1	**2.31**
	
	Os12g43400	hypothetical protein		**2.06**
	
	Os12g43590	FAD binding protein, putative, expressed	AT5G49555.1	**0.50**
	
	Os12g43620	helix-loop-helix DNA-binding domain containing protein	AT4G37850.1	**2.36**
	
	Os12g44010	purple acid phosphatase precursor, putative, expressed	AT2G16430.2	**0.50**
	
	Os12g44070	protein nitrate and chloride transporter, putative, expressed	AT2G39210.1	**2.07**
	
	Os12g44270	glycine-rich cell wall protein precursor, putative	AT3G22800.1	**0.44**
	
	Os12g44310	9,10-9,10 carotenoid cleavage dioxygenase 1, putative, expressed	AT3G63520.1	**2.43**
	
	Os12g44360	sodium/hydrogen exchanger 7, putative, expressed	AT1G14660.1	**5.71**

* - indicates genes common to dehydration stress experiments in rice seedlings of Tyagi *et al*)[48]. Data obtained from the Gene Expression Omnibus (GEO) database at the NCBI website, accession number: GSE 6901

Each QTL described by it's Gramene website QTL accession id, rice map position and the range of genes underlying that QTL.

Interestingly, in two cases the genetic determinants responsible for the QTL phenotype have been linked to a single underlying gene and homologues of these two genes were both present in the list of putative drought-responsive *Musa *transcripts. These two genes are (TIGR: LOC_Os04g23890) and (TIGR: LOC_Os11g03794) underlying QTLs-2 (drought susceptibility, trait symbol AQA045/CQ148) and QTL-5 (cold-tolerance, trait symbol CQP8) respectively. LOC_Os04g23890 encodes for the protein PHOT2 (non-phototrophic hypocotyl 1-like) which is a membrane-bound protein serine/threonine kinase functioning as a blue light photoreceptor in redundancy with PHOT1. PHOT2 is responsible for the trait 'drought susceptibility' and was 2.2-fold up-regulated in response to drought in *Musa*. Both PHOT1 and PHOT2 are thought to help optimize photosynthesis by capturing light energy efficiently and by reducing photodamage [45]. LOC_Os11g03794 encodes for 'Aladin', a structural constituent of ribosomes and is identical to the cDNA 'Huellenlos' (HLL). In wheat, the HLL homologue appears to be involved in plant development, including development of the floral organs [46].

It is tempting when looking at the list of other drought-responsive genes present at these stress-responsive QTLs to try and link gene functions with the overlying trait response, not least because these are potential candidates for explaining the QTL trait, and could therefore be candidates for marker assisted selection programs. Of particular interest at QTL-1 (cold-tolerance) are genes encoding for a Cyt P450 (possibly involved in ABA degradation), and a K^+ ^channel, at QTL-3 (drought-tolerance), a MYB TF (MYB 36) and chaperonins, while at QTL-6 (drought susceptibility) a bHLH protein, a Na/H^+ ^antiporter could clearly have roles in regulating plant drought stress responses. An up-regulated putative carotenoid cleavage dioxygenase (CCD1, TIGR:LOC_Os12g44310) is a member of a gene family believed to be involved in ABA synthesis in *Arabidopsis *and which in rice are associated with the negative regulation of the outgrowth of axillary buds [47]. Clearly such interpretations need to be confirmed through additional experiments including confirmation of gene identities and gene expression analysis. Nonetheless it is interesting to note that 5 of the transcripts common between transcriptomic studies of rice-seedling dehydration stress (see 'Meta-analyses' later), and the *Musa *putative drought stress experiments also colocalize to rice abiotic stress QTLs (table 2), providing further incidental support for the quality of the data and the annotations generated here.

#### Meta-analyses

To date, the only published comparative gene expression study carried out in banana is the work of Santos and co-workers [28], who prepared enriched, full-length cDNA libraries from leaves of the cultivar *M. acuminata *spp. *burmannicodes *var. Calcutta 4, subjected to temperature stress. Fortunately however large-scale studies of the response of the transcriptome to abiotic (drought) stress in other plants species are available e.g. [32-34]. Of particular interest is the work of Tyagi *et al *[48] who examined the responses of rice to abiotic stresses using the Affymetrix Rice Genome Array. In this work, desiccation stress was imposed by drying seedlings between folds of tissue paper for 3 h at room temperature. The expression data sets for the results of these studies were downloaded from the Gene Expression Omnibus (GEO) database at the NCBI website ( accession number: GSE 6901). This data set contained 6,253 genes that were significantly (>2-fold, p < 0.05) differentially expressed in response to desiccation. Despite fundamental differences in the experimental design and in plant developmental status, 330 of the rice seedling transcripts overlappedsignificantly (p = 0.024) with the list of *Musa *putative drought-responsive genes, including 5 that colocalised again with the abiotic stress QTLs outlined in table 2. Of these 330 *Musa *transcripts, 53.4% were up-regulated and 46.6% down-regulated [see Additional file 3].

**Figure 4:**
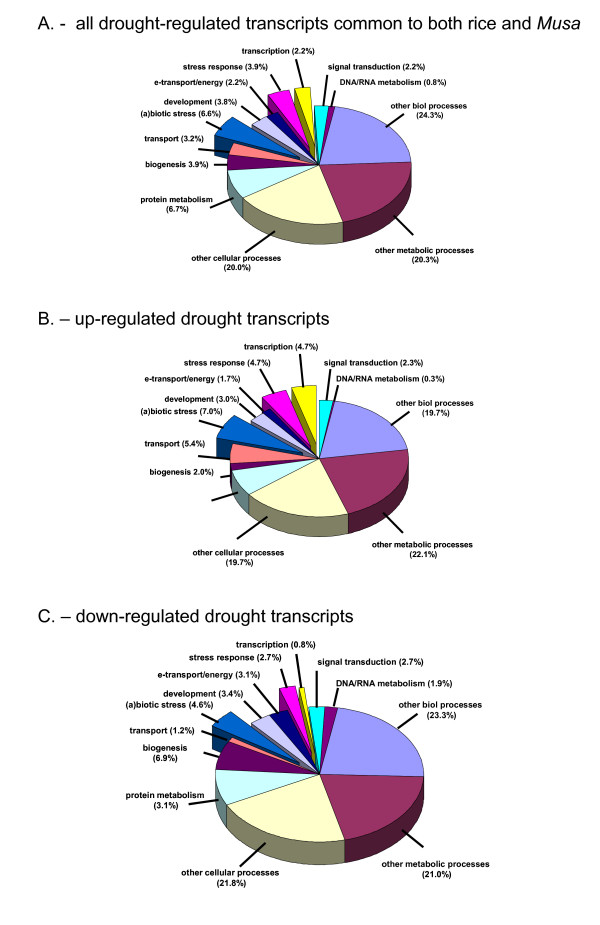
**Overview of the functional classes of the list of 330 dehydration-responsive rice seedling transcripts common to the list of drought-responsive *Musa *transcripts**. Gene annotations and BINS assigned on the basis of *Arabidopsis *gene accession numbers using the TAIR GO website.

297 of the 330 transcripts could further be assigned an *Arabdiopsis *annotation with the HarvEST program, and these annotations were again used to assign functional classifications with the GO software function on the TAIR website. These results are summarised in Figure 4

Looking at the distribution of gene classes we can see that the up-regulated transcripts common to the rice seedling and *Musa *drought-stress experiments, contain proportionally more genes involved in 'transport', 'response to (a)biotic stimulus', 'stress response' and 'transcription', while the classes involved with 'cell biogenesis' and 'electron transport and energy production' are down-regulated. This again agrees with generally-accepted patterns of plant responses to drought/desiccation stress. Within the list of functional genes there is an up-regulation of membrane proteins involved in water transport and osmoregulation, as well as proteins for the detoxification of stress-related reactive oxygen species (ROS) such as glutathione S-transferases, hydrolase, catalase, ascorbate peroxidase etc.. The list of common regulatory genes contains members of the homeodomain proteins (2), pathogen-induced (1), MYB (3), ERF-family (2) and one heat stress factor (HSF) TF with proposed functions again characteristic of TFs involved in regulating plant (a)biotic stress-responses.

#### Comparison with the results of the AtGenExpress

Finally, the list of *Musa *putative drought-responsive transcripts was compared to the results obtained from dehydration stress experiments in *Arabidopsis *carried out as part of the AtGenExpress consortium [34]. In these experiments, dehydration stress was imposed by exposing *Arabidopsis *plants to a dry air stream for 15 minutes on the bench until 10% of their fresh weight was lost [34]. From the list of 518 *Arabidopsis *differentially expressed genes (>2 fold, p < 0.05), 37 (p = 0.06) and 55 (p = 0.003) *Musa *gene homologues overlapped with the drought responsive *Arabidopsis *transcripts from shoot and root tissues respectively. In addition there were 12 *Musa *transcripts common to all 3 data sets (*Musa *severe drought stress, and *Arabidopsis *root and *Arabidopsis *shoot desiccation stress). Therefore despite substantial differences in experimental design, the results of this meta-analysis indirectly support the provisional gene identities assigned to the drought responsive transcripts in *Musa *and illustrate the degree of conservation of the pathways of plant stress responses across plant species, even when they are as distantly related as *Arabidopsis *and *Musa*.

#### Semiquantitative RT-PCR

**Figure 5:**
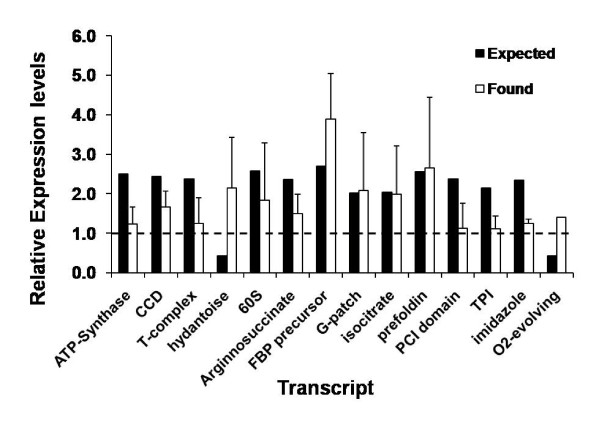
**Overview of the relative transcript abundance of a selection of >2-fold drought-responsive *Musa *transcripts by semi-quantitative RT-PCR**. Primers were designed on the basis of *Musa *unigene sequences displaying highest homology to the rice unigene sequences. Transcript names correspond to the probe set descriptions given in Additional file 3. Results represent the means of 3 individual PCR-amplifications. 'Expected' = Relative expression levels based on microarray data, 'Found' = transcript expression levels as determined by RT-PCR.

The identity of the *Musa *transcripts is based on cross-hybridisation with at least of one 25 bp probe per Rice GeneChip^® ^transcript probe-set. To help confirm the validity of the gDNA probe-based approach and the identity of the differentially-regulated transcripts, primers were designed to a number of drought-responsive transcripts. To do this we used an in-house, proprietary database of *Musa *unigene sequences. For primer design we preferentially used *Musa *sequences which were 'best hit' in a reciprocal BLASTN query of rice unigene sequences (downloaded from the HarvEST website) versus the *Musa *unigene database. In total, primers to 14 sequences were used, and the results from the relative expression levels in control and drought stressed foliar tissue are summarised in Figure 5.

As shown in Figure 5, the RT-PCR results generally showed the same trends, and expression levels generally agreed well with the results from the microarray hybrisation experiments. This has previously been demonstrated in other heterologous cross-hybridisation experiments utilising more closely-related plant species [14,21]. In this work however there were also several cases where the expected *Musa *relative transcript expression values based on the microarray results significantly differed from the RT-PCR results. These discrepancies probably relate to the difficulty in assigning strict functional annotations and designing primers for (*Musa*) transcripts based on homologies to the relatively short oligonucleotide probes of quite distantly-related heterologous species. In addition, the *Musa *unigene collection we used contains only 22,205 unique transcript sequences, which extrapolating from the gene densities of one gene per 4.1 kb reported by Lescot *et al *[26] and a haploid genome size of 560 - 600 Mb [22,23] corresponds to a maximum of only around 16% of the total predicted number of genes present in the *Musa *genome. This means that in some cases it is possible that the 'true' sequence, with highest homology was simply not present in our unigene collection. In support of this, the sequences that produced relative expression results that were not in agreement with the microarray data generally also had lower E-values, or were transcripts derived from large gene families. Undoubtedly the availability of additional EST and genome sequence data will improve the reliability of transcript identification.

## Conclusion

Despite the economic and social importance of *Musa *spp for large sections of the world's population, there have been few systematic studies into banana and plantain responses to abiotic stress [28,49-51] and none to drought. There are also no published genome-wide stress-studies in *Musa *and relatively little sequence data is available particularly when compared to other important crops. Further, despite the large number of drought stress-associated genes identified in both model and non-model plant species, there is still no consensus as to the key processes that determine plant tolerance and survival, and in only a few cases has gene function been defined. Therefore it is likely that the study of bananas and plantains, will result in the identification of additional, novel stress adaptation mechanisms and could represent a powerful resource in the search for plant stress-tolerant genes and/or markers. The aim of this work therefore was to evaluate the use of a heterologous microarray approach to profile the transcriptome of the relatively drought-tolerant *Musa *cultivar 'Cachaco' to chronic drought stress. In contrast to other transcriptomic studies in which short, 'shock-like' treatments have been applied [10,33,52] experimental conditions here were chosen to more closely mimic field conditions and thus to identify genes involved in the long-term adaptation and survival of plants to water deficit.

Cross-hybridisation of *Musa *gDNA to the ATH-1 and Rice GeneChips^® ^showed that the number of probes and probe-sets retained decreased much more rapidly with increasing CDF-values on the ATH-1 array than on the Rice GeneChip^®^. Nonetheless there was still a sufficient degree of homology between *Musa *and *Arabidopsis *to be able to profile a significant proportion of the *Musa *expressed genome using the ATH-1 GeneChip^®^. This would thus tend to support the results of comparative sequence studies in *Musa, Oryza *and *Arabidopsis *that indicate that *Poacea *and eudicot genomes share microsyntenic regions [26,53].

Cross-hybridisation of *Musa *RNA to the Rice GeneChip^® ^identified 2,910 transcripts (probe-sets) displaying a >2-fold difference in expression levels in response to drought. Gene annotations based on rice and *Arabidopsis *databases indicated that many of these transcripts were involved in cellular pathways and processes typically involved in plant (a)biotic stress responses, including a number of genes with TF activity. Importantly, our results share significant overlaps with transcriptome studies in other (model) species, with the list of drought-responsive *Musa *genes including homologues known to be involved in the dehydration stress responses of both rice and *Arabidopsis*. Furthermore, the list also contained a number of transcripts that co-localized to known rice QTLs for both drought and cold responses, including 2 QTLs for which the underlying genetic determinant has been localized to a single (up-regulated) gene. These could therefore be a good target for marker development and could directly incorporated into *Musa *breeding and selection programs. These QTL results further suggest that the *Musa *gene annotations and functionalities assigned on the basis of cross-hybridisation to rice probes are correct and have biological significance. This conclusion is further supported by the results of the RT-PCR results, which largely agreed with the expression results derived from the microarray, except for those cases where homology to available *Musa *unigenes was very low or where multiple gene models were available.

Therefore cross-species (heterologous) microarray studies using gDNA-based probe selection allows the profiling of up to ~58% of the total *Musa *genome despite the absence of substantial sequence data for this species and the large phylogenetic distance from the target species. This is a substantially larger proportion of the transcriptome than has previously reported for this species and as far as we are aware, the largest phylogenetic distance used in a cross-hybridisation study. gDNA probe-based selection thus represents a powerful tool for the study of complex biological responses in a non-model species. While gene ontologies and function of key transcripts have to be carefully validated, the results here underline the potential of this methodology for the identification of (new) mechanisms and pathways of expression control. In the long term this information can lead to the development of new tools and strategies for the development and breeding of important new crop varieties with improved (a)biotic stress tolerance.

## Methods

### Plant Material

All plant material used was obtained as sterile tissue culture from Bioversity's International Transit Center (ITC) located in the Laboratory of Tropical Crop Improvement of Division of Crop Biotechnics, Katholieke Universiteit Leuven, Belgium. These *in vitro *plants were first transplanted into 7.5 litre polyethylene pots containing equal volumes of an autoclaved mixture of enriched commercial peat soil and sand (2:1, v/v), and allowed to grow and stabilise for approximately three months before the onset of the experiment.

### Experimental setup, growth conditions and sampling

Plants were grown at a density of 24 plants per table under a standardised light/dark regime of 12/12 h at 26/23°C respectively with a light intensity of around 10,000 lux (300 watts/m^2^) and a relative humidity of 75%. At the onset of the experiment, all plants were hand-watered until water drained freely from the base of the pots. The control plants were then watered once a day by flooding the tables with a fertigation solution [see Additional file 4] and allowing the pots to stand for 30 min.

Water-deficit (drought) conditions were imposed by withholding water supply for in total up to five weeks. Leaf samples consisted of a 5 - 8 cm wide strip (~3 g fresh weight) removed from one side of the middle of the second, fully expanded leaf down from the top of the plant. Samples were snap frozen in liquid nitrogen and stored at -80°C until analysis. All sampling took place between 14:00 and 17:00 pm. For transcriptome analysis, equal quantities of RNA from 2 replicate plants grown at each time point per treatment were pooled to give a single biological replicate.

### Musa genomic DNA Extraction

Genomic DNA (gDNA) was isolated from young leaves which had been kept in the dark for 48 h to deplete starch and polysaccharide levels using a modified CTAB method essentially as according to Michiels *et al *[54]. Contaminating RNA was removed by addition of 2.5 μl of a 10 μg/ml stock solution of RNase and incubation at 37°C for 30 min. To check the quality and quantity of DNA, 1 μl samples of isolated DNA were run on a 1% agarose gel in TAE buffer (0.04 M Tris-acetate; 1 mM EDTA, pH 8) as outlined by Sambrook *et al *[55]. After staining of the gel with ethidium bromide for 15 min, DNA concentrations were visually estimated by comparison to different amounts of λ-DNA run at the same time. gDNA quality was determined spectrophotometrically using the AU absorbance ratios at 260/280 nm. Samples with a 260/280 ratio of 1.9 - 2.0 were considered as 'pure'.

### Musa RNA Extraction

RNA was extracted using a modified Tris-LiCl method, based on the work of Tattersall *et al *[56]. The modifications involved a DNAse treatment and an additional phenol:chloroform cleanup step. RNA concentrations and purity were determined using a MultiScan Spectrum microtitre plate scanning UV-VIS spectrophotometer (Multiskan Spectrum, Thermolabsystems, Brussels, Belgium). For purity assessment, the AU 260/280 and AU 260/230 ratios were measured and samples with a ratio of ~2.0 were considered as 'pure'. One microgram of total RNA sample was also run on a 1% agarose gel containing ethidium bromide in TAE buffer to check for possible degradation [55]. Gels were imaged using a GelDoc 1000 gel imaging system and Molecular Analyst v1.5 image analysis software version 4 (Bio-Rad, Hercules, CA).

### gDNA Hybridisation and construction of probe mask files

gDNA hybridisation was carried out essentially as described by Hammond *et al *[14]. In brief, *Musa *gDNA was labeled using the Bioprime DNA hybridisation System (Invitrogen) and subsequently hybridised to either the Arabidopsis ATH1 or Rice, Genome Arrays for 16 h at 45 C, using standard Affymetrix hybridisation protocols. This was then followed by the Affymetrix eukaryotic wash protocol that included antibody staining. The GeneChip^® ^Genome Arrays were then hybridised with 0.5 μg of target *Musa *gDNA and scanned on a G2500A GeneArray scanner. Only one gDNA hybridisation was performed, as replicate gDNA hybridisations all challenge the GeneChip^® ^arrays with the whole genome.

From these results a gDNA cell intensity file (.cel file) was generated using the Microarray Analysis Suite software (MAS, v5.0, Affymetrix). This .cel file contains the hybridisation intensities between *Musa *gDNA fragments and all the *A. thaliana *or Rice GeneChip^® ^oligonucleotide probes. The .cel files have been submitted to GEO (Accession ID = GSE16865) and are also available from the NASC Xspecies webpage . Probes showing a suitably strong cross-hybridisation signal were selected from the .cel file using a .cel file parser script (Xspecies v 1.1, available with instructions at ) written in the Perl programming language . This Perl script creates a probe mask (.cdf) file, which is compatible with a range of microarray analysis software packages and provides the template for the generation of a signal across the probe-set when analyzing the test (*Musa*) transcriptome (i.e. the RNA .cel files). Specifically it allows information to be extracted from the RNA .cel files for only those probe-pairs whose perfect-match (PM) probe gDNA hybridisation intensity value is above a user-defined hybridisation intensity threshold (CDF value). In practice the optimal gDNA CDF is determined systematically and empirically using probe masks created with CDF values of between 50 and 1000 (see 'Results'). A probe-set is retained for analysis when it is represented by at least one PM probe-pair per probe-set. Therefore one 25 bp probe, identical in sequence to a *Musa *gDNA fragment is the minimum requirement for inclusion of that probe-set in the subsequent transcriptome analysis of the target *Musa *RNA.

### cRNA Synthesis, Hybridisation and Interpretation of *Musa *transcriptome data using gDNA-based probe selection

The transcriptional responses of the *Musa *cultivar 'Cachaco' to severe drought were determined by challenging rice GeneChip^® ^Genome Arrays with *Musa *foliar RNA, isolated and pooled from two replicate plants subjected to either 3 weeks water-deficit conditions or to control conditions. Approximately 5 μg of *Musa *total RNA was reverse transcribed at 42°C for 1 h to generate first strand cDNA using 100 pmol oligo dT(24) primer containing a 5'-T7 RNA polymerase promoter sequence, 50 mM Tris-HCl (pH 8.3), 75 mM KCl, 3 mM MgCl_2_, 10 mM dithiothreitol (DTT), 10 mM dNTPs and 200 units SuperScript II reverse transcriptase (Invitrogen Life Technologies). Following first strand cDNA synthesis, second strand cDNA was synthesised using 10 units of *Escherichia coli *polymerase I, 10 units of *E. coli *DNA ligase and 2 units of RNase H in a reaction containing 25 mM Tris-HCl (pH 7.5), 100 mM KCl, 5 mM MgCl_2_, 10 mM (NH_4_)SO_4_, 0.15 mM β-NAD+ and 10 mM dNTPs. This second strand synthesis reaction proceeded at 16°C for 2 h before 10 units of T4 DNA polymerase was added and the reaction allowed to proceed for a further 5 min. The reaction was terminated by adding 0.5 M EDTA. Double stranded cDNA products were purified using the GeneChip^® ^Sample Cleanup Module (Affymetrix). The synthesised cDNAs were transcribed *in-vitro *using T7 RNA polymerase (Enzo BioArray High Yield RNA Transcript Labelling Kit, Enzo Life Sciences Inc., Farmingdale, NY, USA) and biotinylated nucleotides to generate biotinylated complementary RNAs (cRNAs). The cRNAs were then purified using the Affymetrix Sample Cleanup Module (Affymetrix) and randomly fragmented at 94°C for 35 min in a buffer containing 40 mM Tris-acetate (pH 8.1), 100 mM potassium acetate, and 30 mM magnesium acetate to generate molecules of approximately 35 to 200 bp. Affymetrix *A. thaliana *ATH1 Genome or Rice Genome Arrays were then hybridised with 15 μg of fragmented, labeled cRNA for 16 h at 45°C as described in the Affymetrix Technical Analysis Manual. GeneChip^® ^arrays were then stained with Streptavidin-Phycoerythrin solution and scanned with an Affymetrix G2500A GeneArray scanner. The Microarray Analysis Suite (MAS Version 5.0; Affymetrix) was used to generate .cel files for each of the RNA hybridisations by scanning and computing summary intensities for each probe without the use of probe mask files. The .cel files have been submitted to GEO (Acession ID = GSE16865) and are also available from the NASC Xspecies webpage . These .cel files were then loaded into the GeneSpring (Agilent Technologies) analysis software package using the Robust Multichip Average (RMA) pre-normalisation algorithm [57]. During .cel file loading and pre-normalisation, .cel files were interpreted using either, (1) the respective *A. thaliana *or rice .cdf files (i.e. with no probe-selection used), or (2) using .cdf files generated from the *Musa *gDNA .cel file with CDF values ranging from 0 to 1000. Following RMA pre-normalisation and masking of individual probes, each probe-set signal value from treated (drought stressed) samples was standardised relative to the probe-set signal value of its corresponding control, to give the relative gene expression ratios between the two conditions.

### Gene ontology's and functional characterisation

Rice and *Arabidopsis *gene annotations (GO ontology's; ) for >2-fold differentially-regulated *Musa *transcripts cross-hybridising to oligonucelotide probes on the Rice GeneChip^®^, were assigned using the NetAffx web tools software  from Affymetrix, or the HarvEST program . Functional classifications of the genes were assigned using the gene ontology (GO) function at the TAIR website , or the GeneSpring software. The significance of these classifications was calculated using a hypergeometric distribution test . The AGI codes of the *Arabidopsis *equivalents the *Musa *transcripts were obtained from the Affymetrix website, and used to allow expression data to be loaded and analysed in MapMan software for visualization of the cellular pathway responses [40,58].

### Microarray meta-analysis

Results were compared with publicly available comparative transcriptome studies of plant abiotic stress responses in rice (*Oryza sativa*) and *A. thaliana*. The list of drought-responsive *Musa *transcripts obtained at a probe mask hybridisation intensity of CDF-550, were compared to the list of *A. thaliana *dehydration stress-responsive genes identified by the AtGenExpress consortium [34] using the MetaAnalyzer tool of Genevestigator [59]. In addition, data from a recent large scale comparative transcriptomics study of the response of rice seedlings to abiotic stress using the Affymetrix Rice Genome Array by Tyagi *et al *is also available [48]. In these studies, desiccation stress was imposed by drying seedlings between folds of tissue paper for 3 h at room temperature. The expression data sets for these studies were downloaded from the Gene Expression Omnibus (GEO) database at the NCBI ( accession number GSE 6901), and the list of differentially expressed genes overlapping with the list of drought responsive *Musa *transcripts identified that were >2 fold different between treated and control samples and using an ANOVA test (p < 0.05). The significance of gene list overlaps was calculated using a hypergeometric distribution test . Finally, the list of differentially drought-regulated *Musa *genes was compared to genes located at known QTLs for drought and other abiotic stresses localized on the physical genetic map of rice. The position of the QTLs were viewed in Gramene  and the list of underlying genes present at each QTL compared to those present in the CDF-550 list.

### Semi-Quantitative PCR

To verify GeneChip^® ^array expression data, semi-quantitative RT-PCR was performed on first strand cDNA prepared from the same 'Cachaco' 3-week, control and drought RNA samples used for the microarray cross-hybridisation experiments. One μg of total RNA from pooled leaf samples derived from 2 individual plants per condition, was used for reverse transcription using the SuperScript II Reverse Transciptase using conditions recommended by the manufacturer (Invitrogen). The cDNA synthesis reaction was carried out using oligo-dT(18) primers (50 μg ml^-1^). PCR amplification of the first strand cDNA was carried out using gene specific primers for a number of transcripts showing >2-fold difference in expression. Primers were designed using an in-house database of *Musa *EST sequences created from available on-line GenBank sequences and a 3' EST database donated to the Global *Musa *Genomics Consortium by Syngenta. From the original 48,445 EST sequences, 33,038 were clustered to give 9,251 contigs which together with the remaining singletons produced a final collection of 22,205 unique *Musa *unigene sequences. To link the *Musa *EST sequences with the transcripts identified by cross-hybridisation to the Rice GeneChip^® ^microarray, a local BLAST search of the *Musa *unigene sequences versus the entire rice Unigene set (Assembly 1.09), downloaded from the HarvEST website was carried out at E-value stringency settings of both 10 and 0.1. In addition rice Unigene sequences corresponding to the differentially drought-regulated Probe-sets were BLASTed against the *Musa *unigene dataset. Primers for semi-quantitative PCR were designed to the cDNA sequences of those *Musa *gene homologues localized within the QTLs for abiotic stress responses in rice as well as a *Musa *Actin-1 control transcript (GenBank accession AF285176). Primers were designed using the Primer3 primer design tool [60];  and initial primer sequences were checked for secondary structures using the netprimer program, . PCR reactions were carried out using 25 μl per reaction consisting of 2 ng of cDNA sample, 1 μM of 5'- and 3'-primer and 7.5 μl of master mix. The reaction conditions were 94°C (2 min) for one cycle, and then 94°C (30 s) and 55°C (30 s) and 72°C (30 s), for 24 - 32 cycles, before a final extension of 72 C for 10 minutes. Transcript levels of each gene were normalized to *Musa *Actin-1, and the expression of each gene expressed relative to the expression in control plants [see Additional file 5].

## Abbreviations

gDNA: genomic DNA; cel: cell intensity files; cdf: probe mask layout description files; CDF: hybridisation intensity threshold value; EST: Expressed Sequence Tag; GO: Gene Ontology; PM: Perfect Match; BIN: functional gene category code.

## Authors' contributions

MWD - experimental design, plant maintenance, DNA and RNA isolation, primer development, RT-PCR and data analysis, manuscript preparation and revisions, NSG - gDNA and RNA microarray hybridisations, data extraction and analysis, BV - EST analysis, bioinformatics, RS - provision of plant materials and experimental design, STM - perl script development, manuscript editing, JK - manuscript editing and revision. All authors read and approved the final manuscript

## Supplementary Material

Additional file 1 **Davey, Table 1 - List 2-fold drought-responsive genes.** List of genes >2-fold differentially regulated in response to 3-weeks chronic drought stress in *Musa *variety 'Cachaco', using a gDNA-based hybridisation intensity threshold value (CDF value) of 550. Click here for file

Additional file 2 **Davey, Table 2 - MapMan mappings for ATH drought homologues.** List of the MapMan mappings for available *Arabidopsis *homologues of the list of drought-responsive *Musa *transcripts. Click here for file

Additional file 3 **Davey, Table 3 - List common drought responsive genes**. List of the 330, >2-fold drought-responsive genes common to *Musa *3-week chronic drought stress experiment and rice seedling dehydration stress experiment (GEO GSE6901 Rice drought). Differentially expressed *Musa *transcripts identified using a hybridisation intensity threshold value (CDF value) of 550. Click here for file

Additional file 4 **Davey, Table 4 - fertigation solution**. Table detailing the composition of the fertigation solution used for the growth of plants used in this experiment. Click here for file

Additional file 5 **Davey, Table 5 - RT-PCR transcripts and primers**. Subset of drought-responsive *Musa *genes, annotations and relative expression levels as checked by semi-quantitative RT-PCR. Click here for file
