# Supplementary material for: Heterologous oligonucleotide microarrays for transcriptomics in a non-model species; a proof-of-concept study of drought stress in Musa
Source: BMC Genomics. 2009 Sep 16;10:436. doi: 10.1186/1471-2164-10-436 (PMC2761422; doi:10.1186/1471-2164-10-436)
Supplement: Additional file 4 — Davey, Table 4 - fertigation solution. Table detailing the composition of the fertigation solution used for the growth of plants used in this experiment. [file 1471-2164-10-436-S4.doc]

Davey *et al*, Additional file 4 - Nutrient contents of the fertigation solution used for the growth of *Musa* plants used in this experiment

| **Macroelements** | **concentration (mM)** | **microelements** | **concentration (µM)** |
| --- | --- | --- | --- |
| K | 7 | Fe | 15 |
| Mg | 1.5 | Mn | 10 |
| Ca | 5 | Cu | 0.5 |
| NO3 | 12.5 | Zn | 4 |
| H2PO4 | 1.5 | Bo | 20 |
| SO4 | 2.5 | Mo | 0.5 |
